# Identification of High-Risk Individuals for Osteoporosis and Fragility Fractures in Cushing’s Syndrome: A Promising Predictive Approach

**DOI:** 10.3390/jcm15062442

**Published:** 2026-03-23

**Authors:** Enes Ucgul, Burak Menekse, Ogulcan Boz, Huseyin Demirci, Bekir Ucan, Erman Cakal, Takako Araki, Muhammed Kizilgul

**Affiliations:** 1 Department of Endocrinology and Metabolism, Ankara Etlik City Hospital, 06170 Ankara, Türkiye; drburakmenekse@gmail.com (B.M.); ogulcanboz@gmail.com (O.B.); drhdemirci@gmail.com (H.D.); uzm.dr.bekir@hotmail.com (B.U.); ermancakal@hotmail.com (E.C.); muhammedkzgl@gmail.com (M.K.); 2 Division of Diabetes, Endocrinology and Metabolism Department of Medicine, University of Minnesota, Minneapolis, MN 55455, USA; taraki@umn.edu

**Keywords:** Cushing’s syndrome, osteoporosis, fragility fracture, cortisol, diabetes mellitus, risk prediction model

## Abstract

**Background**: Cushing’s syndrome (CS) causes excessive cortisol exposure, leading to significant skeletal complications. However, there is no validated, CS-specific model to predict osteoporosis and fracture risk. This study aimed to identify independent predictors and develop a practical clinical scoring system. **Methods**: A retrospective study was conducted on 139 patients with CS diagnosed between 2014 and 2025. Demographic, clinical, and biochemical data were analyzed. Osteoporosis was defined using dual-energy X-Ray absorptiometry criteria. Logistic regression analyses identified independent predictors, and the Cushing-Related Osteoporosis Risk Estimation (CORE) Score was constructed from normalized beta coefficients of significant variables. **Results**: Osteoporosis was present in 35.9% and fragility fractures in 13.4% of patients. Independent predictors included age ≥ 51 years, symptom duration ≥ 13.5 months, diabetes mellitus, late-night salivary cortisol ≥ 0.42 μg/dL, and midnight serum cortisol ≥ 10.25 μg/dL (all *p* < 0.05). The CORE Score (0–6 points) showed strong diagnostic performance for osteoporosis (AUC 0.827; sensitivity 88%, specificity 72%) and fractures (AUC 0.866; sensitivity 84%, specificity 78%). Each one-point increase in the CORE Score elevates the risk of osteoporotic fracture by 3.13 times (*p* < 0.001). **Conclusions**: The CORE Score represents a promising disease-specific tool for early identification of CS patients at increased risk of osteoporosis and fragility fractures, enabling more personalized management and follow-up strategies, such as prioritizing bone-protective interventions and closer skeletal monitoring. Early identification of high-risk individuals may also facilitate timely therapeutic interventions, potentially reducing future fracture risk.

## 1. Introduction

Cushing’s syndrome (CS) is a rare endocrinological disease. The primary causes of CS are excessive steroid production or excessive exogenous glucocorticoid use. The most common cause of CS is Cushing’s disease (60–70%), which originates from a pituitary adenoma, followed by adrenal tumors (20–30%), and rarely ectopic sources from non-pituitary tumors (10–15%) [1,2,3]. CS presents with a wide range of clinical features. Specific features include facial plethora, easy bruising, and purple striae. Moreover, CS can cause complications such as hypertension, hyperglycemia, neurocognitive problems, and immunosuppression [4,5].

Continuous exposure to cortisol can also negatively impact bone health. This adverse effect of cortisol impairs osteoblast differentiation and stimulates apoptosis of both osteoblasts and osteocytes [6,7]. Furthermore, cortisol has an indirect negative impact on bone metabolism through hypogonadism, reduced insulin-like growth factor-1 secretion and action, pulsatile parathyroid hormone secretion, excessive weight gain, and sarcopenia. This entire process results in decreased bone formation, increased bone resorption, and an increased risk of osteoporotic fracture [8].

In several studies, osteoporosis was reported in approximately 28–50% of CS. In these studies, dual-energy X-Ray absorptiometry (DXA) is the most commonly used method for detecting osteoporosis [9,10]. Additionally, studies investigating factors associated with osteoporosis in patients with CS have identified several key factors, including the duration of excess cortisol exposure, midnight cortisol levels, and 24 h urinary free cortisol levels (24 h UFC) [10,11,12]. Although numerous studies have been conducted to assess risk factors for osteoporosis, the current literature remains inconclusive, specifically regarding which parameters increase the risk of osteoporosis in patients with CS, the reliability of DXA in this population, and the identification of high fracture risk [13]. FRAX, the Garvan tool, and Qfracture are used to estimate the risk of osteoporotic fractures in the general population; however, none have been specifically validated for patients with CS [10,13]. Moreover, these tools do not incorporate disease-specific determinants, such as cortisol burden, circadian rhythm disruption, or metabolic complications, such as diabetes mellitus, which may substantially influence bone quality and fracture susceptibility in CS. Because of this, there is still a need for an accessible and practical model that can accurately identify individuals among CS early on who are at increased risk of osteoporosis and fractures.

This study aims to develop a practical scoring system to identify individuals with CS at a higher risk of osteoporosis and fractures early, and to determine which patients need closer monitoring to prevent fractures.

## 2. Methods

### 2.1. Study Population

**Figure 1:**
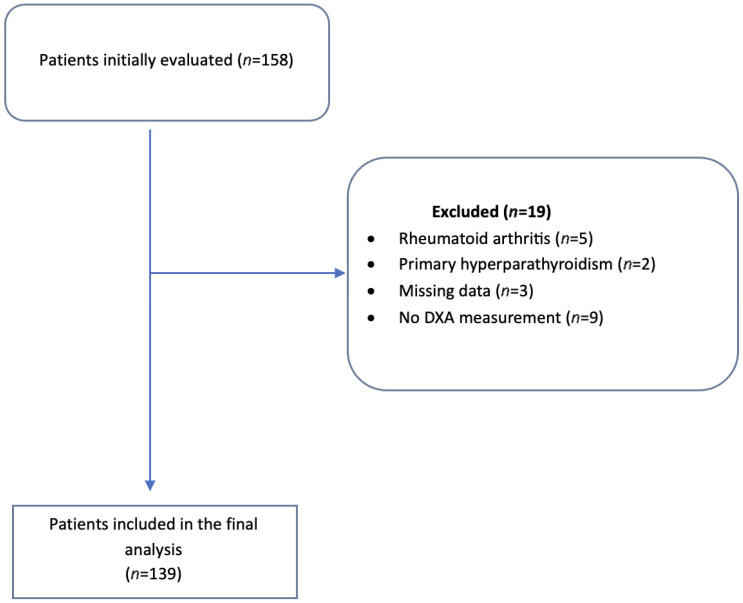
Patient enrollment and exclusion flowchart.

This retrospective study included patients diagnosed with CS between January 2014 and January 2025 at Etlik City Hospital, Dışkapı Yıldırım Beyazıt Training and Research Hospital, and the University of Minnesota Hospital (Study number: #00005168). The diagnosis of CS was confirmed based on clinical, biochemical, and radiological criteria, following the latest international guidelines [14]. Patients aged 18 and older with bone mineral density (BMD) measurements taken at diagnosis and showing biochemical and clinical signs of CS were included. They were classified into subtypes: pituitary, adrenal, and ectopic CS according to the underlying cause of hypercortisolism. Exclusion criteria consisted of incomplete medical records, a prior diagnosis of osteoporosis, comorbid conditions, and any history of diseases or medications that significantly affect bone metabolism (such as rheumatoid arthritis, primary hyperparathyroidism, thyroid disorders, chronic kidney disease, and diabetic patients taking pioglitazone), as well as missing essential diagnostic information (midnight serum and late-night salivary cortisol measurements or DXA results at diagnosis). From an initial group of 158 patients (Figure 1), 5 were excluded due to rheumatoid arthritis, 2 for primary hyperparathyroidism, 3 for missing midnight serum and late-night salivary cortisol data, and 9 for lacking DXA measurements at diagnosis. Therefore, 139 patients were included in the final analysis.

Additionally, histories of osteoporotic fractures over the past 11 years were systematically gathered from patient records, including vertebral, hip, and other low-trauma fracture types associated with decreased bone strength. All study procedures adhered to the Declaration of Helsinki, and ethical approval was granted by the local Ethics Committee (approval number/date: [AEŞH-BADEK2-2025-111/20-05-2025]). Due to the study’s retrospective nature, informed consent was waived.

### 2.2. Data Collection

Demographic data (age, gender, body mass index [BMI]), clinical features (duration of symptoms, presence of hypertension or diabetes mellitus, and physical findings associated with CS such as buffalo hump, moon face, striae, and central obesity), biochemical parameters, and history of osteoporotic fractures were retrospectively gathered from electronic medical records.

The biochemical evaluation included measurements of basal serum cortisol, ACTH, results of the 1 mg and 2 mg overnight dexamethasone suppression tests (DST), midnight serum cortisol, late-night salivary cortisol, 24 h UFC, dehydroepiandrosterone sulfate (DHEA-S), and 25-hydroxyvitamin D (25-OH vitamin D) levels.

Late-night salivary cortisol, midnight serum cortisol, basal serum cortisol, ACTH, DHEA-S, and 25-OH vitamin D levels were measured using the electrochemiluminescence immunoassay method with the Cobas e 801 analyzer (Roche Diagnostics, Mannheim, Germany). The 24 h UFC levels were measured using the radioimmunoassay method with the Berthold Gamma Counter B 2111 Multi Crystal system (Berthold Technologies, Bad Wildbad, Germany).

Bone Mineral Density (BMD) was measured using DXA with a Fujifilm device (FDX Visionary-DR, Fujifilm, Tokyo, Japan). Osteoporosis was defined as a T-score of ≤−2.5 in individuals aged 50 years and older. For participants under 50, a Z-score of ≤2.0, combined with a history of osteoporotic fracture or an underlying condition that causes secondary osteoporosis, was used [15].

### 2.3. Statistical Analysis

Continuous variables were reported as mean ± standard deviation (SD) or median with interquartile range (IQR), based on the distribution evaluated by the Shapiro–Wilk test. Categorical variables were summarized using counts and percentages. Comparisons between osteoporotic and non-osteoporotic groups used Student’s *t*-test or the Mann–Whitney U test for continuous variables, and the chi-square test or Fisher’s exact test for categorical variables, as appropriate. Initially, univariate logistic regression analyses identified variables associated with osteoporosis. Optimal cut-off values for age, symptom duration, and midnight serum cortisol were determined through receiver operating characteristic (ROC) curve analysis by identifying the points that maximized Youden’s Index. The cut-off for late-night salivary cortisol was adopted from the laboratory reference value. These dichotomized variables were subsequently used as predictors in the univariate and multivariate logistic regression analyses. Variables with *p* < 0.05 in univariate analyses were considered for multivariate modeling. Additionally, clinically significant covariates identified a priori were forced into the model regardless of their univariate significance to ensure proper adjustment for potential confounders. The results were presented as odds ratios (OR) with their corresponding 95% confidence intervals (CI), beta coefficients (B), and *p*-values.

### 2.4. Developing the Cushing-Related Osteoporosis Risk Estimation (CORE) Score

Based on the multivariate logistic regression results, a risk-scoring system, the Cushing-Related Osteoporosis Risk Estimation (CORE) Score, was developed. A risk-scoring system was developed using the statistically significant variables identified in the multivariate logistic regression model. The beta coefficient for the diabetes mellitus (DM) variable was chosen as the reference point, as it had the least significant and independent contribution to the risk of osteoporosis. All other beta coefficients were divided by this value to obtain normalized beta values. To improve clinical usefulness and clarity, these normalized values were grouped into point intervals. Specifically, variables with normalized beta values between 1.0 and 1.4 were assigned 1 point, while those with values of 1.4 or higher were assigned 2 points. Based on this method, the total score ranged from 0 to 6, with higher scores indicating a greater predicted risk of osteoporosis. Afterwards, the relationship between this risk score and the presence of osteoporotic fractures was analyzed using both univariate and multivariate logistic regression methods. The discriminative ability of the CORE Score was evaluated using ROC curve analysis. The area under the curve (AUC) was determined to assess diagnostic performance. Reported values included sensitivity, specificity, AUC, and Youden’s Index. Also, Model calibration was evaluated by the Hosmer–Lemeshow goodness-of-fit test (*p* > 0.05 indicating good agreement between predicted and observed risk). All statistical analyses were conducted using IBM SPSS Statistics version 27, and *p*-values < 0.05 were considered statistically significant.

## 3. Results

### 3.1. Patient Characteristics

The baseline demographic and clinical characteristics of patients with CS are summarized in Table 1. Our study included 64 patients with pituitary CS, 70 with adrenal CS, and 5 with ectopic Cushing’s syndrome. It included more cases of adrenal CS than pituitary CS. Among the osteoporotic group, 8 were men and 42 were women, whereas in the non-osteoporotic group, 13 were men and 76 were women, with no statistically significant difference in gender distribution (*p* = 0.826). The mean age was significantly higher in osteoporotic patients compared to non-osteoporotic patients (55 ± 11.8 years vs. 47 ± 12.2 years, *p* < 0.001). Median symptom duration was longer in osteoporotic patients (13 months [IQR: 8–24]) compared to non-osteoporotic patients (10 months [IQR: 6–13.00]) (*p* = 0.004).

Biochemically, median midnight serum cortisol and late-night salivary cortisol levels were significantly higher in osteoporotic patients (15 μg/dL and 0.48 μg/dL, respectively) compared to non-osteoporotic patients (10 μg/dL and 0.205 μg/dL, respectively) (both *p* < 0.001). There were no statistically significant differences between the groups in BMI, 1 mg DST cortisol levels, 2 mg DST cortisol levels, or 24 h UFC levels (all *p* > 0.05).

### 3.2. Prevalence of Osteoporosis by CS Subtype

**Figure 2:**
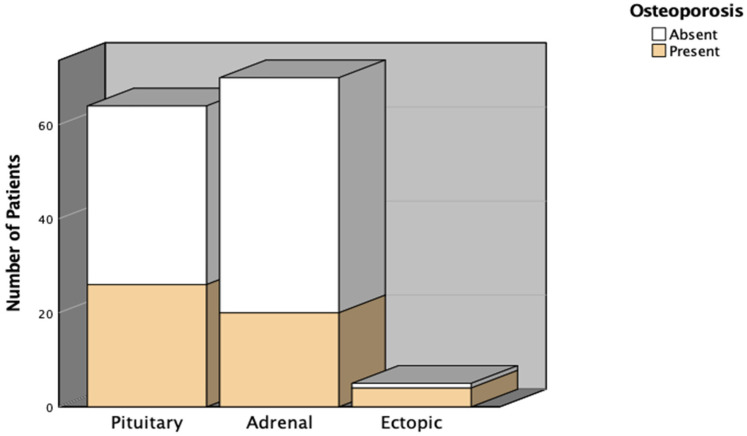
Distribution of Osteoporosis Among Cushing’s Syndrome Subtypes.

Table 2 and Figure 2 display the distribution of osteoporosis across different CS subtypes. Osteoporosis was present in 40.6% of patients with pituitary CS, 28.6% with adrenal CS, and 80% with ectopic CS. Overall, 35.9% of the study population had osteoporosis. Regarding osteoporotic fractures, 12 patients (18.8%) in the pituitary group, 6 patients (8.6%) in the adrenal group, and 1 patient in the ectopic group experienced fractures. The total osteoporotic fracture rate was 13.7%, with no significant difference among subtypes (*p* = 0.211).

### 3.3. Results of Predictive Factors for Osteoporosis in CS

Prior to univariate logistic regression, the optimal cut-off points for age, symptom duration, and midnight serum cortisol were identified through ROC analyses and the Youden index. The predefined laboratory reference cut-off was used for late-night salivary cortisol. The selected thresholds are shown in Table 3.

In univariate logistic regression analysis (Table 4), factors significantly associated with osteoporosis included late-night salivary cortisol ≥ 0.42 μg/dL (OR: 4.342, 95% CI: 2.114–8.920, *p* < 0.001), midnight serum cortisol ≥ 10.25 μg/dL (OR: 9.402, 95% CI: 3.635–24.315, *p* < 0.001), symptom duration ≥ 13.5 months (OR: 5.328, 95% CI: 2.427–11.699, *p* < 0.001), age ≥ 51 years (OR: 2.611, 95% CI: 1.315–5.184, *p* = 0.006), presence of diabetes mellitus (OR: 4.604, 95% CI: 2.147–9.874, *p* < 0.001), presence of hypertension (OR: 3.436, 95% CI: 1.301–9.079, *p* = 0.013), basal cortisol level (OR: 1.048, 95% CI: 1.003–1.095, *p* = 0.035), and BMI (OR: 0.911, 95% CI: 0.844–0.984, *p* = 0.017). Variables such as gender, 25-OH Vitamin D, 24 h UFC level, and 1 mg DST cortisol levels were not significantly associated with osteoporosis.

Multivariate logistic regression analysis (Table 5) identified age ≥ 51 years (OR: 6.68, 95% CI: 1.76–25.35, *p* = 0.005), symptom duration ≥ 13.5 months (OR: 10.38, 95% CI: 2.73–38.84, *p* < 0.001), diabetes mellitus (OR: 5.22, 95% CI: 1.50–18.12 *p* = 0.009), late-night salivary cortisol ≥ 0.42 μg/dL (OR: 5.30, 95% CI: 1.46–19.20 *p* = 0.011), and midnight serum cortisol ≥ 10.25 μg/dL (OR: 5.81, 95% CI: 1.46–23.03 *p* = 0.012) as independent predictors of osteoporosis. However, gender, hypertension, basal cortisol level, 24 h UFC level, and 25-OH Vitamin D level are not independent significant factors. Additionally, Multicollinearity was assessed using the Variance Inflation Factor (VIF) obtained from a linear regression model including all predictor variables. All VIF values were <3 and tolerance values were >0.2, indicating no evidence of multicollinearity among predictors.

### 3.4. CORE Score Predicts Osteoporosis and Osteoporotic Fracture Risk in CS Within Our Cohort

In our multivariate logistic regression model identified 5 statistically significant variables (Table 5). Using this, we generated a risk scoring system and shown in Table 6. The beta coefficient of the Diabetes Mellitus (DM) variable (B = 1.653) was used as the reference point, as it had the least significant and independent contribution among the variables. All other coefficients were divided by this value to derive normalized beta values. Based on these normalized values, variables were assigned as follows: values between 1.0 and 1.4 received 1 point, while those equal to or greater than 1.4 were given 2 points. The maximum total score ranged from 0 to 6.

**Figure 3:**
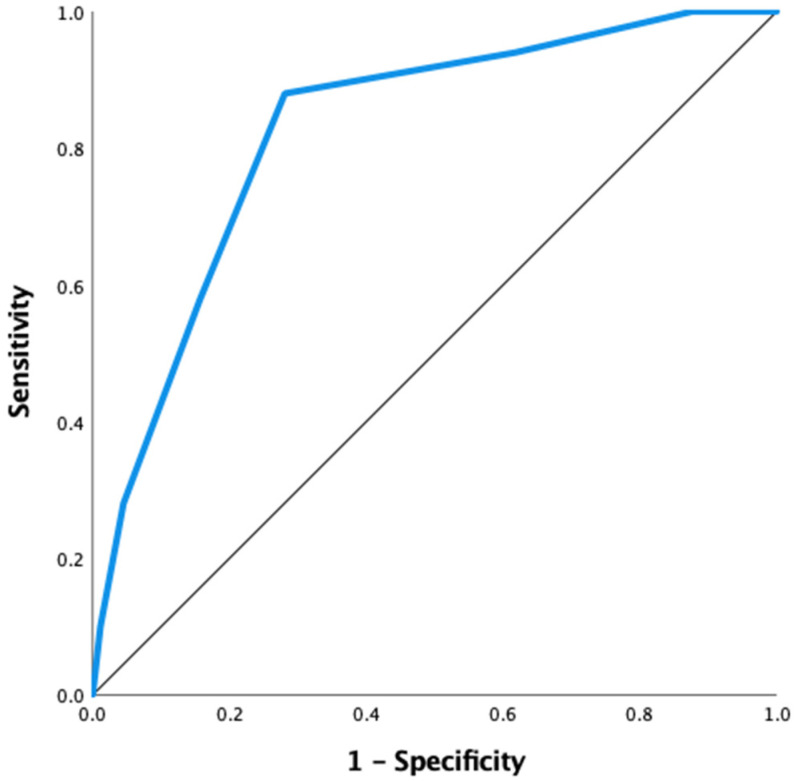
ROC Curve for the CORE Score in Osteoporosis Prediction.

ROC curve analysis was conducted to find the optimal cut-off value for the CORE Score in predicting osteoporosis. The model yielded an AUC of 0.827, indicating good discriminative ability. The optimal cutoff was 2.5, with a sensitivity of 88% and a specificity of 72%. The Youden’s Index at this point was 0.631, indicating a strong balance between sensitivity and specificity. Also, at the selected cut-off value, the CORE Score demonstrated a positive predictive value of 91.4% and a negative predictive value of 97.3% for identifying osteoporosis. These results suggest that the CORE Score is a reliable predictor, effectively identifying patients with osteoporosis while maintaining an acceptable false-positive rate. The ROC curve for the CORE Score is illustrated in Figure 3.

**Figure 4:**
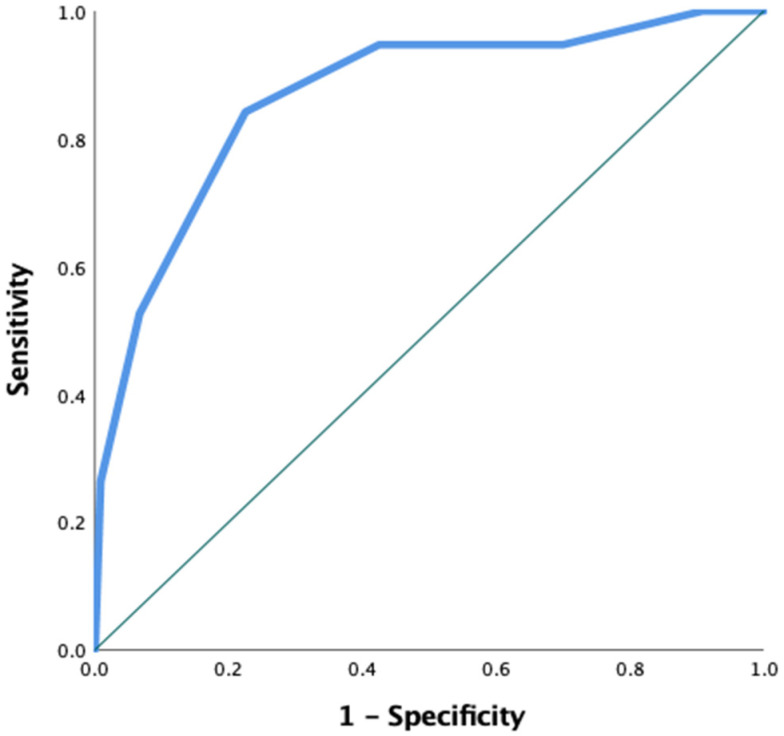
ROC Curve of the CORE Score for Osteoporotic Fracture Prediction.

ROC curve analysis was repeated to assess the ability of the CORE Score to distinguish osteoporotic fractures. The analysis showed an AUC of 0.866, indicating excellent diagnostic accuracy. The optimal cut-off value was 3.5, yielding a sensitivity of 84% and a specificity of 78%. These results suggest that the CORE Score is a reliable tool for identifying patients at higher risk of osteoporotic fractures, as shown in Figure 4. Additionally, the Hosmer–Lemeshow goodness-of-fit test indicated no evidence of poor calibration (χ^2^ = 7.56, df = 4, *p* = 0.109), suggesting good agreement between predicted and observed osteoporosis risk.

The results of predictive factors for osteoporotic fracture, including all univariate and multivariate logistic regressions, are shown in Table 7. The CORE Score was significantly associated with the risk of osteoporotic fracture in both the univariate model (*p* = 0.003) and the multivariate model (*p* < 0.001). The CORE Score remained a strong and independent predictor in the multivariate model (OR: 3.13, 95% CI: 1.91–5.13, *p* = <0.001). Other variables, including gender, 24-UFC, BMI, 1 mg DST, and 25-OH Vitamin D, were not significantly associated with fracture risk in either model. Furthermore, the fracture prediction model demonstrated excellent calibration, with no evidence of lack of fit (Hosmer–Lemeshow χ^2^ = 3.76, df = 4, *p* = 0.440).

## 4. Discussion

Osteoporosis is one of the well-known complications of CS, yet the risk assessment system has not been well developed. Our research examined clinical and biochemical predictors of osteoporosis and fractures in patients with CS and proposes a new risk assessment system: the CORE Score. To our knowledge, this is the first scoring system specifically developed to predict which patients are at higher risk of osteoporosis and fragility fractures in CS.

We found that 5 factors—longer symptom duration (≥13.5 months), age ≥51 years, presence of diabetes mellitus, and higher levels of both midnight serum cortisol (≥10.25 μg/dL) and late-night salivary cortisol (≥0.42 μg/dL)—were significantly linked to an increased risk of osteoporosis. Notably, these variables remained independent predictors in the multivariate analysis, highlighting their importance in clinical risk assessment. The CORE Score demonstrated a high sensitivity of 88%, and a relatively good specificity of 72%. Furthermore, each one-point increase in the CORE Score was associated with a 3.13-fold increase in the risk of osteoporotic fractures in patients with CS. This important finding emphasizes the score’s potential usefulness not only for predicting osteoporosis but also for assessing fracture risk in these patients with CS.

In our cohort, the prevalence of osteoporosis was higher in patients with Cushing’s disease (40.6%) than in those with adrenal CS (28.6%), although the difference was not statistically significant. Rahaman et al. also found no differences in BMD or osteoporosis frequency among CS subtypes [16]. In contrast, Nariko et al. reported a higher prevalence of osteoporosis in those with adrenal Cushing’s syndrome than in those with pituitary Cushing’s disease [17]. Methodological difference in bone density evaluation may cause this discrepancy. Nariko et al. assessed BMD solely in the lumbar spine, included a smaller patient cohort than ours, and did not include symptom duration. Furthermore, there is a significant difference in BMI between the two groups, with the Pituitary Cushing’s group having a higher BMI, unlike our research, which found no significant differences between groups. The literature indicates that a higher BMI is associated with better BMD [17].

Additionally, the increased use of abdominal imaging in our clinical practice may lead to earlier detection of adrenal lesions, potentially resulting in the diagnosis of adrenal CS at an earlier stage, before significant bone loss occurs. In our study, neither gender nor body mass index was significantly associated with osteoporosis or osteoporotic fractures in patients with CS. However, the literature shows inconsistent findings on this matter. For example, a previous study of patients with CS (pituitary origin, n = 68; adrenal origin, n = 28) regarding osteoporotic fractures found that male patients had a lower risk of fractures compared to female patients [18]. In contrast, another study has reported that male patients may have a higher risk of developing osteoporosis and fragility fractures [19]. These inconsistencies may stem from differences in gender distribution and sample size across studies.

Furthermore, BMI is often reported in the literature as a significant factor affecting osteoporosis risk in the general population, typically with higher BMI considered protective [20,21]. We did not observe a significant difference in baseline BMI between patients with and without osteoporosis. In our cohort, BMI showed only a modest association with osteoporosis in univariate analysis, but this association lost significance after multivariate adjustment. This may be related to the generally high BMI values in patients with CS, which may limit its discriminative power within this population.

In the present study, we showed that age over 51 years is a significant risk factor for osteoporosis. This age also aligns with the typical onset of menopause in women and andropause in men [22,23] and an independent risk factor of osteoporosis. This finding aligns with several reports in the literature. For example, Amodru et al. demonstrated an inverse relationship between age and BMD in CS. Although they did not specify a particular age cutoff, the authors classified their patients as either younger than 65 years or 65 years and older, based on WHO criteria [24]. On the other hand, some studies have produced conflicting results. Libuse et al., for example, did not find a significant association between age and osteoporosis [25]. Nevertheless, a closer examination of their methodology suggests that the absence of a clear age difference between the study groups may have limited the ability to identify such an association. The importance of age as a key risk factor may stem from the cumulative effects of aging-related conditions, such as sarcopenia, decreased physical activity, and hypogonadism, which are known to contribute to bone loss and higher fracture risk [8]. Additionally, the present study showed that DM is a significant independent factor. This situation may be explained by the fact that DM has been linked to decreased bone quality through mechanisms including the accumulation of advanced glycation end products in bone collagen, diminished osteoblast function, and changes in bone microarchitecture, which increase fracture risk independent of bone mineral density [26].

Late-night salivary cortisol levels (≥0.42 μg/dL) and midnight serum cortisol levels (≥10.25 μg/dL) are independent and significant predictors of osteoporosis in patients with CS. These findings align with previous research. Multiple studies have demonstrated that midnight serum cortisol levels correlate with reduced bone mineral density, and late-night salivary cortisol is a reliable marker of disease activity and severity, which are the primary determinants of osteoporosis risk in this population [27,28,29,30]. In contrast, morning cortisol levels show significant overlap between patients and controls, making them less useful for assessing disease severity or bone risk [28,29]. Dexamethasone-suppressed cortisol and 24 h urinary free cortisol are useful for diagnosis; however, their association with bone complications is less robust and more variable, as they may not fully capture the loss of circadian rhythm or intermittent hypercortisolism [12,30]. In the literature, Trementino et al. reported that disease duration and midnight cortisol levels significantly correlated with decreased BMD in the lumbar spine (L1–L4). Additionally, disease duration has been identified as a predictive factor for fracture risk [27]. In another study assessing salivary cortisol levels in postmenopausal women from the OsteoLaus cohort, higher evening salivary cortisol levels were associated with lower trabecular bone scores, an indicator of compromised bone microarchitecture, underscoring the importance of hypercortisolism in the deterioration of bone quality and the increased risk of fractures [31]. One possible explanation for this connection is that prolonged and elevated hypercortisolism impairs osteoblast function, induces osteocyte apoptosis, and boosts osteoclast activity, resulting in increased bone resorption and decreased bone formation. Additionally, chronic cortisol excess may lead to myopathy, growth hormone deficiency, and hypogonadism, all of which are known to negatively impact bone quality and increase fracture risk [6,8].

In this study, the proposed CORE Score provides a simple, evidence-based tool to identify CS patients at increased risk for osteoporosis and osteoporotic fractures. A CORE Score >2.5 for osteoporosis and >3.5 for fractures indicates higher risk and should prompt closer follow-up and further evaluation. In these high-risk patients, especially when DXA is normal despite strong clinical suspicion, additional techniques such as trabecular bone score or quantitative CT should be considered [32,33]. To date, no validated scoring system specifically designed to predict osteoporosis risk and osteoporotic fracture risk in patients with CS has been reported in the literature. The CORE Score, therefore, may address a critical gap by offering a straightforward yet effective clinical tool that combines risk factors, such as age, disease duration, cortisol levels, and diabetes, to assess osteoporosis and osteoporotic fracture risk in CS patients. It enables earlier detection of individuals at high risk who might otherwise be missed by traditional BMD measurements alone, enabling more focused follow-up and timely treatment.

This study has several notable strengths, including the inclusion of a relatively large and well-characterized cohort of patients with CS, the availability of comprehensive clinical and biochemical data at the time of diagnosis, and the development of a simple, clinically applicable risk-scoring system with good discriminative performance. Nevertheless, certain limitations must be acknowledged.

First, the retrospective single-cohort design may limit the external validity and generalizability of the findings. Given the rarity of CS, all eligible patients were included in the model derivation process to enhance statistical robustness and facilitate the development of a disease-specific risk prediction tool. Accordingly, the CORE Score should be considered a preliminary derivation model that requires validation in independent, preferably prospective and multicenter cohorts before routine clinical implementation is advisable. Second, although internal model calibration was formally assessed and showed acceptable agreement between predicted and observed outcomes, as assessed by the Hosmer–Lemeshow goodness-of-fit test, external validation was not feasible at this stage. Ongoing patient recruitment at participating centers is expected to enable future validation studies to confirm the model’s reproducibility and broader applicability. Third, markers reflecting bone microarchitecture, such as trabecular bone score, were not systematically available due to the retrospective design, which may have limited the assessment of skeletal fragility beyond bone mineral density measurements.

## 5. Conclusions

This study identified several key and independent factors associated with osteoporosis and the risk of osteoporotic fractures in patients with CS. The primary contributors include prolonged exposure to excess cortisol, advanced age, disruption of the physiological circadian rhythm, and DM. Late-night salivary and midnight serum cortisol are more strongly associated with osteoporosis in CS than morning cortisol, dexamethasone-suppressed cortisol, or 24 h UFC. Importantly, the CORE Score may serve as a practical tool for endocrinologists, enabling early identification of individuals at higher risk for osteoporosis and osteoporotic fractures. If DXA cannot be used, the CORE Score may help physicians identify patients at higher risk and those who need close monitoring.

Furthermore, our results suggest that the CORE Score is a promising method for assessing osteoporotic fracture risk in patients with CS. It can assist clinicians in identifying patients who are more prone to fractures and therefore require closer observation and proactive treatment. While the CORE Score demonstrates encouraging predictive ability, it is essential to validate it in larger, prospective studies to confirm its reliability and effectiveness across a broader range of clinical contexts.

## Figures and Tables

**Table 1:** Baseline Demographic and Clinical Characteristics of the Study Population.

Variable	Osteoporotic Patients	Non-Osteoporotic Patients	*p*-Value
Gender, (*n*)			0.826
Man	8	13	
Woman	42	76	
Age, years (mean, SD)	55 ± 11.8	47 ± 12.2	<0.001
Duration of symptoms, months (median, IQR)	13 (8–24)	10 (6–13)	0.004
BMI, kg/m^2^ (median, IQR)	31 (29.3–33.2)	31.3 (29.3–35.2)	0.541
Basal cortisol, μg/dL (median, IQR)	21 (18–25)	15.85 (12–21.25)	<0.001
2 mg DST cortisol, μg/dL (median, IQR)	10 (5.45–14.1)	6.2 (3.65–12.3)	0.072
1 mg DST cortisol, μg/dL (median, IQR)	11 (6.2–17)	9.15 (4.90–14.15)	0.199
24 h UFC (<45 μg/24 h) (median, IQR)	92 (65–260)	94.5 (52–166.25)	0.729
Midnight serum cortisol (<7.5 μg/dL) (median, IQR)	15 (12–18.4)	10 (7.75–15.92)	<0.001
Late-night salivary cortisol, (<0.42 μg/dL) (median, IQR)	0.48 (0.20–0.825)	0.205 (0.15–0.46)	<0.001
DHEA-S, μg/dL (median, IQR)	112 (29.5–166)	88 (24–193)	0.837
25-OH vitamin D, ng/mL (median, IQR)	12 (9.2–17)	16 (12–22)	0.016
Diabetes mellitus (%)	74%	38.2%	<0.001
Hypertension (%)	85.7%	67.1%	0.010

Abbreviations: BMI: Body Mass Index; DST: Dexamethasone Suppression Test; IQR: Interquartile Range; SD: Standard Deviation; DHEA-S: Dehydroepiandrosterone Sulfate; 25-OH vitamin D: 25-Hydroxyvitamin D; 24 h Urinary Free Cortisol: 24 h UFC.

**Table 2:** Subtype-Based Analysis of Osteoporosis, Fracture Rates, and Demographic Variables in Cushing’s Syndrome.

Cushing’s Syndrome Subtype	Osteoporotic Patients *n* (%)	Non-Osteoporotic Patients *n* (%)	Age (Mean ± SD)	Gender (*n*) (F/M)	Osteoporotic Fracture *n* (%)
Pituitary	26 (40.6%)	38 (59.4%)	49 ± 12.4	58/6	12 (18.8%)
Adrenal	20 (28.6%)	50 (71.4%)	51 ± 13	58/12	6 (8.6%)
Ectopic	4 (80%)	1 (20%)	53 ± 12.2	2/3	1 (20%)
Total	50 (36%)	89 (64%)	-	139	19 (13.7%)

**Table 3:** Cut-off Points, Sensitivity, and Specificity for Clinical Predictors by ROC curve analysis.

Variable	Optimal Cut-Off	Sensitivity (%)	Specificity (%)
Age	51	46.3	84.3
Symptom Duration	13.5 months	50	76.4
Midnight Serum Cortisol	10.25 μg/dL	87.5	57.3
Late-night salivary Cortisol	0.42 μg/dL	62.5	75.6

**Table 4:** Univariate Logistic Regression Analysis of Factors Associated with Osteoporosis.

Variable	Odds Ratio (Exp(B))	95% CI (Lower–Upper)	*p*-Value
Gender (male ref.)	0.898	0.345–2.341	0.826
Late-night salivary cortisol ≥ 0.42 μg/dL	4.342	2.114–8.920	<0.001
Midnight serum cortisol ≥ 10.25 μg/dL	9.402	3.635–24.315	<0.001
Symptom duration ≥ 13.5 months	5.328	2.427–11.699	<0.001
Age ≥ 51 years	2.611	1.315–5.184	0.006
Hypertension	3.436	1.301–9.079	0.013
Diabetes Mellitus	4.604	2.147–9.874	<0.001
Basal cortisol level, μg/dL	1.048	1.003–1.095	0.035
24 h UFC, μg/24 h	1.000	1.000–1.001	0.367
Body Mass Index	0.911	0.844–0.984	0.017
1 mg DST cortisol, μg/dL	1.026	0.990–1.064	0.162
25-OH vitamin D, ng/mL	0.959	0.911–1.010	0.115

Abbreviations: DST: Dexamethasone Suppression Test; 25-OH vitamin D: 25-Hydroxyvitamin D; 24 h Urinary Free Cortisol: 24 h UFC.

**Table 5:** Multivariate Logistic Regression Analysis of Independent Predictors of Osteoporosis.

Variable	B	Odds Ratio (Exp(B))	95% Confidence Interval (Lower–Upper)	*p*-Value
Gender (male ref.)	0.378	1.45	0.36–5.88	0.595
Body Mass Index	−0.06	0.99	0.87–1.12	0.929
Age ≥ 51 years	1.899	6.68	1.76–25.35	0.005
Symptom duration ≥ 13.5 months	2.340	10.38	2.73–38.84	<0.001
Diabetes Mellitus	1.653	5.22	1.50–18.12	0.009
Hypertension	0.514	1.67	0.37–7.40	0.499
Late-night salivary cortisol ≥ 0.42 μg/dL	1.670	5.30	1.46–19.20	0.011
Midnight serum cortisol ≥ 10.25 μg/dL	1.760	5.81	1.46–23.03	0.012
Basal cortisol level, μg/dL	0.024	1.02	0.97–1.08	0.347
25-OH vitamin D, ng/mL	−0.086	0.92	0.84–1.01	0.073

Abbreviations: 25-OH vitamin D: 25-Hydroxyvitamin D.

**Table 6:** Predictive Scoring Model for Osteoporosis: Variable Weights and Assigned Points.

Risk Factor	Beta Coefficient (B)	Normalized B (B/1.653)	Assigned Points
Late-night salivary cortisol ≥ 0.42 μg/dL	1.670	1.01	1
Midnight serum cortisol ≥ 10.25 μg/dL	1.760	1.06	1
Symptom duration ≥ 13.5 months	2.340	1.41	2
Diabetes Mellitus	1.653	1.00	1
Age ≥ 51 years	1.899	1.14	1

Points were assigned based on beta coefficients from the multivariate model. Coefficients were normalized using the smallest significant coefficient (diabetes mellitus) and categorized into predefined intervals to construct an additive risk score.

**Table 7:** Univariate and Multivariate Logistic Regression Analysis of Factors Associated with Osteoporotic Fracture.

Variable	B	OR (95% CI)	*p* (Univariate)	B	OR (95% CI)	*p* (Multivariate)
Gender (male ref.)	1.28	3.6 (0.45–28.53)	0.225	0.873	3.93 (0.46–33.90)	0.213
CORE Score	1.16	3.20 (1.94–5.29)	0.003	1.016	3.13 (1.91–5.13)	<0.001
24 h urinary free cortisol (μg/24 h)	0.000	1.00 (0.99–1.00)	0.998	-	-	-
Body mass index (kg/m^2^)	−0.87	0.92 (0.81–1.03)	0.151	-	-	-
1 mg DST (μg/dL)	0.014	1.01 (0.97–1.05)	0.473	-	-	-
25-OH Vitamin D, (ng/mL)	−0.060	0.94 (0.85–1.04)	0.241	-	-	-

Abbreviations: CORE: Cushing-Related Osteoporosis Risk Estimation; DST: Dexamethasone Suppression Test; 25-OH vitamin D: 25-Hydroxyvitamin D.

## Data Availability

The datasets generated and analyzed during the current study are available from the corresponding author upon reasonable request.

## Abbreviations

The following abbreviations are used in this manuscript:

CS	Cushing Syndrome
CORE	Cushing-Related Osteoporosis Risk Estimation
DXA	Dual-energy X-Ray Absorptiometry
24-h UFC	24 h Urinary Free Cortisol Level
DST	Dexamethasone Suppression Test
DHEA-S	Dehydroepiandrosterone Sulfate
25-OH vitamin D	25-hydroxyvitamin D
BMD	Bone Mineral Density
ROC	Receiver Operating Characteristic
DM	Diabetes Mellitus
AUC	Area Under The Curve
OR	Odds Ratios
CI	Confidence Intervals
B	Beta Coefficients
